# Praziquantel Treatment of *Schistosoma mansoni* Infected Mice Renders Them Less Susceptible to Reinfection

**DOI:** 10.3389/fimmu.2021.748387

**Published:** 2021-12-10

**Authors:** Justin Komguep Nono, Thabo Mpotje, Paballo Mosala, Nada Abdel Aziz, Fungai Musaigwa, Lerato Hlaka, Thomas Spangenberg, Frank Brombacher

**Affiliations:** ^1^ Division of Immunology and South African Medical Research Council (SAMRC) Immunology of Infectious Diseases, Faculty of Health Sciences, University of Cape Town, Cape Town, South Africa; ^2^ International Centre for Genetic Engineering and Biotechnology (ICGEB), Cape Town Component, Cape Town, South Africa; ^3^ Laboratory of ImmunoBiology and Helminth Infections, Institute of Medical Research and Medicinal Plant Studies, Ministry of Scientific Research and Innovation, Yaoundé, Cameroon; ^4^ Chemistry Department, Faculty of Science, Cairo University, Giza, Egypt; ^5^ Global Health Institute of Merck, Ares Trading S.A., a Subsidiary of Merck KGaA Darmstadt Germany, Eysins, Switzerland; ^6^ Wellcome Centre for Infectious Diseases Research in Africa (CIDRI-Africa) and Institute of Infectious Disease and Molecular Medicine (IDM), Faculty of Health Sciences, University of Cape Town, Cape Town, South Africa

**Keywords:** schistosomiasis, praziquantel, infection-treatment cycles, reinfection, protective immunity

## Abstract

Beyond transient control of the infection, additional benefits of mass drug administration of praziquantel in endemic communities have been suggested in communities but not mechanistically investigated experimentally. The present study sought to evaluate the additional and hitherto unreported benefits of repeated mass drug administration of praziquantel. We used a tractable mouse model of *Schistosoma mansoni* infection to assess the effects of repeated infection-treatment cycles on the host susceptibility to reinfection. Parasitaemia was assessed by quantification of Schistosoma egg burden in liver tissues and morbidity was followed up by histological observation of liver lesions by microscopy and using biochemical measurement of liver transaminases. Immune responses were further determined by serum probing of schistosoma-specific antibodies, cytokines and quantification of liver cellular and soluble mediator responses by flow cytometry and ELISA, respectively. At similar ages and comparable gender distribution, groups of mice undergoing higher number of infections treatment cycles over a longer period, remained susceptible to reinfection by the parasite, as judged by the presence of eggs and the associated increasing pathology in the liver tissues. However, notably, there was a clear and significantly higher propensity to lower egg burden upon reinfection when compared to counterparts undergoing a lower number of infection-treatment cycles. This relative reduction of susceptibility to infection was paralleled by a more robust humoral response against parasite antigens, elevated serum IL-4 and liver cytokines. Of note, praziquantel treatment of infected mice left them at a higher baseline of serum IL-4, IgE and liver cytokines but lower CD4+ T cell -derived cytokines when compared to infected non-treated mice supporting an immunological treatment-induced advantage of previously infected mice over naïve mice and infected/not treated mice. Notably, repeated infection-treatment cycles did not preclude the infection-driven aggravation of collagen deposition in the livers over time and was corroborated by a more robust local production of inflammatory cytokines in the most exposed livers. Taken together, our data reveal that treatment of *S. mansoni*-infected hosts with praziquantel rewires the immune system to a conformation less permissive to subsequent reinfection in mice. Provided the data are translatable from mouse to human, our findings may provide mechanistic support to the potential benefits of more frequent MDAs in high transmission areas to allow rapid acquisition of protective immunity against reinfection.

## Introduction

Schistosomiasis heavily cripples and kills many, predominantly in Africa (1–3). As no vaccine or drug is available to prevent infection, the mainstay of morbidity control in endemic communities is a yearly treatment with praziquantel (PZQ) *e.g.* mass drug administration programs (MDA) to school-aged children (4). The World Health Organization (WHO) recommends a single dose treatment with PZQ (40 mg/kg) that provides cure rates of 76.4% for *S. mansoni* infection (1, 5). A considerable progress has been made since then relying on this strategy (2), building more momentum for the community to address the next step of disease elimination. The new goal of schistosomiasis elimination have been set and several bodies of evidence are advocating for the higher benefits of multiple treatment rounds with PZQ in endemic areas (6, 7).

To support potential upcoming clinical studies and strategies on the field, the *S. mansoni* infected mouse model offers a unique possibility to assess in an accelerated view the development of schistosomiasis infection that could take decades to unfold in clinical settings (8–10). Although inherently resistant to the course of repeated cycles of reinfection as a result of the portal shunting of eggs (11), the mouse model still closely recapitulates some features of human schistosomiasis infections such as the anatomo-pathologic and pathophysiologic features of the infection including the immunological specifics and when used with a single inbred strain, all similarly prone to portal shunting, differences in resistance to infection could be reliably assigned to additional immunological changes (12). In fact, the model has been used extensively in the characterization of *in vivo* efficacy of drug candidate against the parasite (13). Here, we evaluated the susceptibility to subsequent reinfection under repeated cycles of infection followed by PZQ treatment. The key areas of focus in this study were the alterations in egg burden (as a proxy for susceptibility to infection) and specific immune responses prompted by PZQ treatment of infected mice.

## Materials and Methods

### Mice and Ethics

The use of inbred adults (8-10 weeks old of 20-30g), wild-type and immunocompetent animals only in this study, *i.e.* C57BL/6, Balb/C and 129SV mice, was approved by the University of Cape Town Animal Ethics Committee (Protocol 016/027) and all animal experiments were performed in accordance with the South African National Standard (SANS 10386:2008), and consistent with the 3Rs guiding principles of reduction, refinement and replacement. Specifically, for the present study on mouse infection with *Schistosoma mansoni*, animals were monitored daily with the weight and signs of distress reported. Specific signs were diarrhoea, weakness, slowness of gate, isolation with scores assigned to each parameter. Practically, mice that lose 20% of their initial body weight and/or show aggravated weakness i.e. unable to grip firmly on the cage and/or presenting with bloody diarrhoea apparent on the animal fur or the simultaneous occurrence of 2 out of the 3 following symptoms i.e. “10-19% weight loss”, “loss of grip power” or “bloody diarrhoea for 24-47h”, were euthanized immediately. For euthanasia, mice were killed *via* prolonged halothane inhalation (> 5 minutes), the fatal anaesthesia was confirmed by a total absence of pedal reflex (toe pinch) and eye blink reflex as well as the total absence of a respiratory rate. The mice were then completely exsanguinated by cardiac puncture and cervical dislocation was carried out to ensure complete euthanasia prior to removal of tissues.

### *Schistosoma mansoni* Infection

*Biomphalaria glabrata* snails (a gift from Adrian Mountford, York, UK) and NMRI female mice were used to maintain and expand the *S. mansoni* parasite larvae. Anaesthetised mice were percutaneously infected by exposition of their shaved abdomen for 30 minutes to live *S. mansoni* cercariae (20 to 35, depending on experimental set-ups) in snail culture water. Cercariae from our study are distributed by BEI Resources, (NIAID, NIH, USA), *passaged in B. glabrata*, through infection of NMRI mice, strain NR-21962.

### Preparation of PZQ Solution and Administration Schemes

For the oral treatment of animals, PZQ (Merck KGaA, Darmstadt Germany) was weighted at quantities sufficient for 400 mg/kg of animal body weight into suitable graduated and labelled containers, a dose experimentally shown to be highly efficacious in clearing worms (13). For the preparation, 10 parts 70% Tween/30% Ethanol (EtOH) were added and mixed using a magnetic stirrer. Next, 90 parts of distilled sterile water were slowly added. Stirring was continued until a homogeneous suspension was obtained. Formulations were kept under magnetic stirring until the end of each treatment. The suspensions were administered within 3 hours of preparation. The volume of administration to mice was 200 µL. *S. mansoni*-infected mice were administered by oral gavage twice during week 6 post infection for deparasitization, as previously optimized in mice (14). Deparasitized animals were further subjected or not to cycles of reinfection and deparasitization with PZQ up to a maximum total of 3 infection cycles for some animals. Basically, in a first series of experiments to assess the impact of repetitive cycles of infection with *S. mansoni* cercariae and treatment with PZQ, C57BL/6 mice were set into three groups i.e.

i) the first group of animals were kept for a total of 25 weeks and singly infected at week 17 (termed group 1°) after having being treated (sub-group B) or not with PZQ (sub-group A) at week 14 prior to infection to assess the differential impact of PZQ treatment in naïve mice on response to subsequent infection
ii) the second group of animals also kept for a similar 25 weeks long treated with PZQ at week 6, infected for the first time at week 8, further treated with PZQ at week 14 to finally undergo a final 6 week long infection at week 17 (group 2°) and finally
iii) the third group of animals also kept for a maximum of 25 weeks infected at week, treated with PZQ at week 6, reinfected at week 8, treated again with PZQ at week 14 then finally reinfected at week 17 (termed group 3°).

Animals from all groups were euthanized at week 15 (time point 1 i.e. P1) or week 25 (time point 2, i.e. P2) for assessment of parasitological, immunological and pathological changes between groups.

In a second series of experiments to assess the impact of infection with *S. mansoni* cercariae and that of treatment with PZQ on the immune responses of mice, C57BL/6 mice were into three groups i.e.:

i) A group of naïve mice kept for 16 weeks without infection or treatment
ii) A group of mice kept for 16 weeks and infected a week 0 with no treatment with PZQ thereafter
iii) A group of mice kept for 16 weeks, infected with *S. mansoni* cercariae at week 0 and treated with PZQ (400 mg/kg twice within a week)

In this second series of experiments all animals were sacrificed at week 16 and parasitological and immunological parameters were analysed.

### Sampling (Liver, Serum)

*S. mansoni*-infected and deparasitized animals were euthanised 8 weeks after their final cycle of infection-treatment and exsanguinated. Blood was collected and centrifuged in serum separator tubes (BD Bioscience, San Diego, CA) at 8 000×g for 10 min at 4°C to retrieve the serum. The upper aqueous serum phase was aliquoted into tubes and stored at -80°C until further use. Livers were excised then approximately one-quarter of each liver was used for cell isolation and flow cytometric analyses as previously described (14, 15), another quarter was stored in formalin for histology, a quarter used for egg count and the last quarter used to estimate the relative tissue cytokine contents as a proxy for the local response elicited by trapped parasite eggs.

### Serum Analyses

#### Antibody Titers

*S. mansoni* antigen-specific serum antibody isotypes IgG1and IgE from infected mice were determined as previously described (15). Briefly, the flat-bottom 96-well plates were coated with 10 µg/ml of *Schistosoma* egg antigen (SEA), blocked with 2% (w/v) milk powder for 2 h at 37°C and serum samples were loaded and incubated overnight at 4°C. Alkaline phosphatase labelled secondary antibody was added and incubated for 2 h at 37°C. The plates were developed by addition of 4-nitrophenyl as a substrate (Sigma). The absorbance was read at 405 nm using VersaMax microplate spectrophotometer (Molecular Devices, Germany).

#### Liver Enzymes

Hepatocellular damage was assessed by measuring the serum levels of alanine and aspartate transaminases at the National Health Laboratory Service of South Africa (Cape Town) as per the reported procedures (12, 15).

#### Cytokine ELISA

Quantities of IL-4, IL-10, IL-13, IL-17, TGF-β, TNF-α and IFN-γ in collected sera were measured by sandwich ELISA as previously described (15).

### Flow Cytometric Analyses of Liver Cells

Single cell suspensions from the liver tissue were stained for surface markers with antibody mixes of the following antibodies: F4/80 (PE-Cy7, clone BM8) from Affymetrix eBiosciences; CD4 (PerCP-Cy 5.5, clone RM4-5); CD11b (V450, clone M1/70); CD23 (PE, clone B3B4), IL-4 (A488, clone 11B11), IL-13 (PE-Cy7, clone eBio13A), IL-10 (PE, clone JES5-16E3), IFN-g (AF647, clone XMG1.2), Arginase-1 (PE, cat IC5868P, Lot ADBB0516121); SiglecF (APC-Cy7, clone E50-2440); Lineage (PerCP-Cy5.5, clones 145-2C11, which recognizes Mouse CD3e; M1/70, which recognizes CD11b; RA3-6B2, which recognizes CD45R/B220; TER-119, which recognizes Ly-76, mouse erythroid cells; and RB6-8C5, which recognizes Ly-6G and Ly-6C.); B220 (V500, clone RA3-6B2) from BD Biosciences and T1/ST2 (FITC, clone DJ8) from MDBiosciences. Antibodies were diluted in FACS buffer (1X PBS with 1% BSA and 0.1% NaN3 + 2% inactivated Rat serum and 2% α-FcγII/III (clone 2.4G2), to avoid nonspecific binding, and the cocktails used to stain cell suspensions (1x10^6^) for 30 min on ice. Stained cells were then washed with PBS containing 0.1% BSA (Roche, Switzerland) and 0.1% NaN_3_. For intracellular cytokine staining, cells were restimulated with a cocktail of PMA/Ionomycin/Monensin for 4h at 37°C then fixed in 2% PFA, permeabilized and cytokine production was analysed as previously described (15). Acquisition was conducted using BD LSR Fortessa, and data analysis was performed with FlowJo software (Treestar, Ashland, OR, US).

### Histology

Liver samples were fixed in neutral buffered formalin, cut into 5–7 μm sections. The obtained sections were stained with hematoxylin and eosin (H & E). Stained sections were scrutinized for trapped eggs and/or surrounding granulomas to confirm effective infection. Granuloma diameter of 20-50 granulomas per animal was determined using an ocular micrometer (Nikon NIS-Elements, Nikon Corporation, Tokyo, Japan). For fibrosis assessment, tissue sections were stained with chromotrope 2R and aniline blue solution (CAB) and counterstained with Wegert’s hematoxylin for collagen staining. The fraction of liver tissue covered with the blue deposition of collagen was determined automatically by computer-aided microscopy (Nikon NIS-Elements, Nikon Corporation, Tokyo, Japan). All microscopical assessments were performed by blinded operators, only made aware of codes provided for each group of slides. The unmasking was performed by another team of operators before analyses to avoid any bias in the microscopy-aided assessments.

### Liver Egg Burden Determination

Excised pieces of liver were used to count schistosome eggs after digestion in 4% KOH_aq_ for 18 h, as previously described (15).

### Tissue Homogenate and Cytokine ELISA

Liver tissue samples were homogenised using extraction buffer (1X PBS buffer with 2 μg protease inhibitor, and 0.1% Tween), centrifuged and the supernatant was collected for ELISA assays. Protein content was measured using BCA assay according to manufacturer’s instructions (Thermo Scientific, Catalog number 23225). Quantities of IL-4, IL-10, IL-13, IL-17, TGF-β, TNF-α and IFN-γ in liver homogenates were measured by sandwich ELISA as previously described (15).

### Statistical Analysis

GraphPad Prism 6 software (http://www.prism-software.com) was used for analyses. Values were displayed as mean ± SEM and differences between groups were tested for significance, with each statistical test used specified in the corresponding figure legend. Values of p<0.05 were considered statistically significant.

## Results

### Repeated Cycles of *S. mansoni* Infections and PZQ Treatments Render Mice Less Susceptible to Further Reinfection With *S. mansoni*

**Figure 1:**
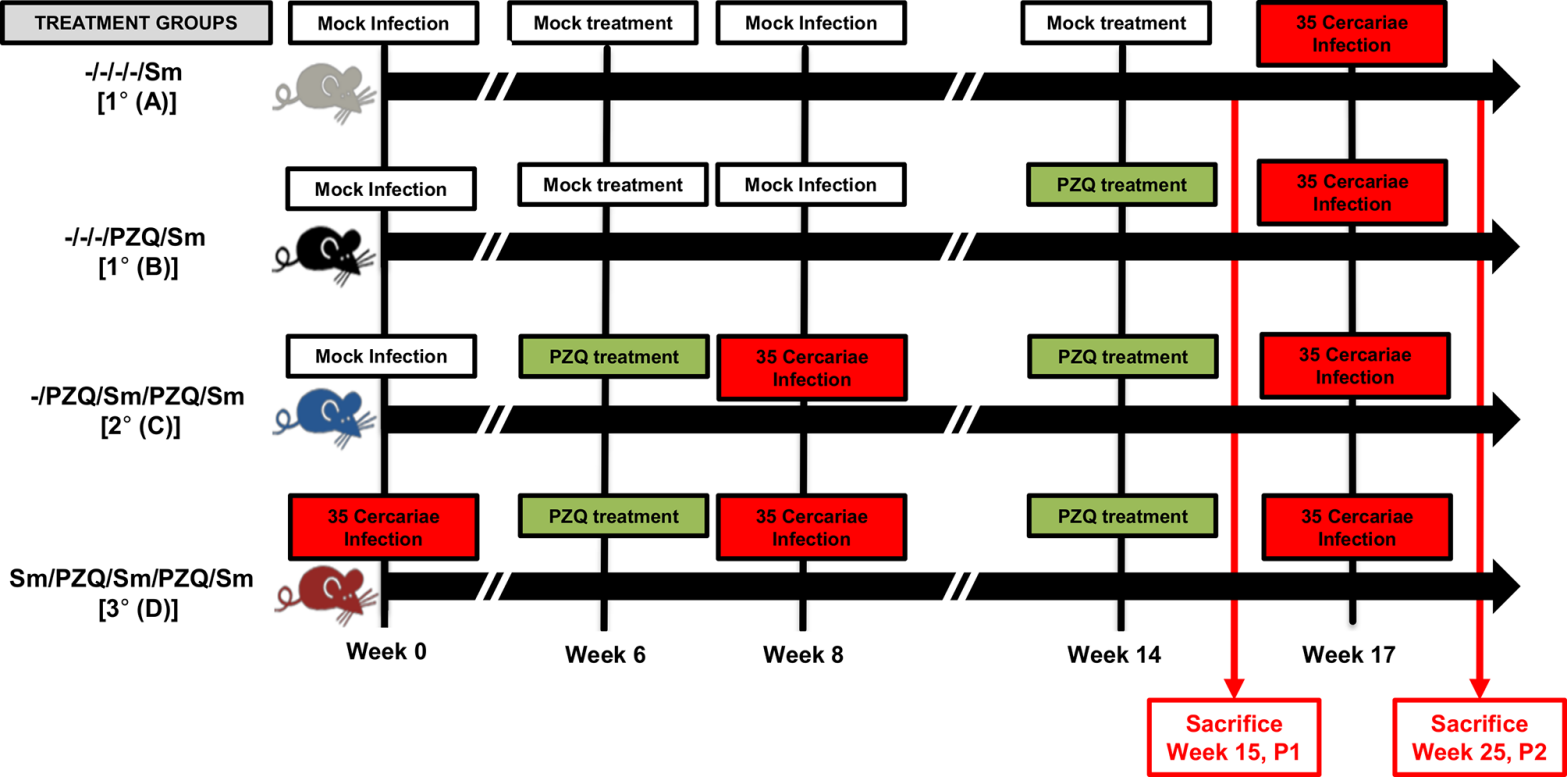
Adjusted study design. Following experimental infection of mice with *S. mansoni* cercariae and anti-parasitic treatment with racemic Praziquantel (PZQ), the susceptibility to reinfection with *S. mansoni* and schistosomiasis-driven pathology was assessed. Briefly, C57BL/6 wild-type and immunocompetent mice (8-10 weeks old to ensure the use of adult mice only) of 20-30g were separated into four experimental groups omitting long term infections without interspersed episodes of PZQ treatments *i.e.* groups A, B, C and D. Group A for an overall total of 1 infection and no PZQ treatment *i.e.* -/-/-/-/Sm, group B with a single pretreatment with PZQ and an overall total of 1 infection *i.e.* -/-/-/PZQ/Sm; group C for a single pretreatment with PZQ, an overall total of two infections intercalated by another treatment with PZQ *i.e.* -/PZQ/Sm/PZQ/Sm; group D for an overall total of three infections intercalated by two PZQ treatments *i.e.* Sm/PZQ/Sm/PZQ/Sm, as indicated on the illustration. Practically, mice were infected percutaneously with 35 *S. mansoni* cercariae (or cercariae water as Mock) and treated 6 weeks later with racemic PZQ (or carrier solution) by administration at 400mg/kg twice by oral gavage. One week following the end of anti-parasitic treatment with PZQ, mice were reinfected percutaneously with 35 *S. mansoni* cercariae (or cercariae water as Mock) *i.e.* at week 8. Animals were then treated with racemic PZQ (or carrier solution) 6 weeks later *i.e.* at week 14 by daily oral administration of PZQ at 400mg/kg by oral gavage for 7 days. Two weeks after the end of this second round of anti-parasitic treatment *i.e.* at week 17, all mice, including controls from group A and B, were reinfected percutaneously, with 35 *S. mansoni* cercariae. Animals were sacrificed in part at week 15 *p.i.* (time point 1 i.e. P1) or week 25 (time point 2, *i.e.* P2) for assessment of parasitological, immunological and pathological changes between groups. Specifically, biopsies of spleen, liver and blood were done for laboratory analyses of egg burdens, Antibody ELISA, Cytokine ELISA, Histology and Flow cytometry. Two independent experiments were conducted with 4-5 mice per group to be sacrificed at P1 (Group A &B, 4 mice; Group C, 5 mice; Group D, 4 mice) and 5-7 mice per group to be sacrificed at P2 (Group A, 5 mice; Group B, 4 mice; Group C, 6 mice; Group D, 7 mice). A total of 70 mice were used for this scheme.

The impact of repeated cycles of schistosomiasis infection-treatment with PZQ on the host was assessed. To do so, C57BL/6 mice were either repeatedly infected with 35 cercariae of *S. mansoni* or mock-infected with carrier water (snail water) and treated *p.o.* (*per os* i.e. orally) twice within a week period with 400 mg/kg of PZQ or vehicle to equate either one (1°), two (2°) or three (3°) rounds of infections separated by treatments (**Figure S1**). Consistent with our own past observations, control groups of animals infected over an entire follow-up period of more than 18 weeks without treatment, initially planned to be considered (**Figure S1**), were then omitted due to their reported premature death in our settings (**Figures S2A–C**), opposing the ethical requirements of reduction and refinement on animal welfare. When assessing a lower parasite inoculum size of 20 cercariae to circumvent premature death of mice over our follow up period of 25 weeks, we noted a strong likelihood of failed infections in our settings at this inoculum size (**Figure S2D**). Only other groups of animals under the different counts of infection and treatment cycles were therefore included in our final experimental design with an efficient low infectious dose of 35 cercariae (**Figure 1**), further surveyed and monitored for changes in body and organ weights over time (**Figure S3**). No noticeable body weight differences were observed between any groups. Fifteen or twenty-five weeks after the first infection (**Figure S3**), animals were sacrificed, and spleens and livers retrieved for determination of the weight index (**Figures S3B–E**). From the early endpoint of week 15 (P1), a clear and robust trend of hepato and splenomegaly was apparent in infected mice when compared to naïve mice, though no difference in spleen or liver weights was observed between animals from the groups of different numbers of infection-treatment cycles.

**Figure 2:**
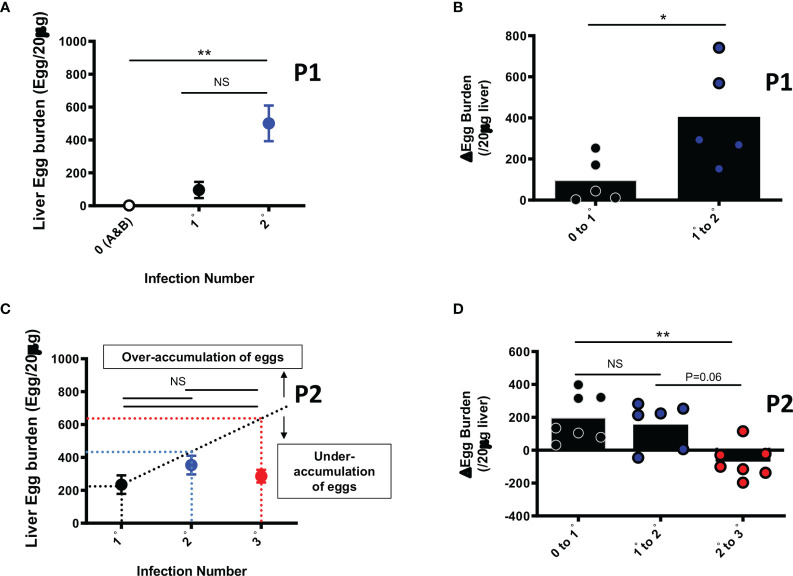
Liver Egg burdens after repeated cycles of *S. mansoni* infection and PZQ treatments. Outcome measure = Egg accumulation rate in liver **(A)** Liver Egg burdens are displayed for mice after first (1°) and second (2°) infection cycle with 35 *S. mansoni* cercariae and analysed at week 15 *i.e.* P1. **(B)** Liver egg burden changes in animals undergoing their first (0 to 1°) or second (1° to 2°) cycle of infection intercalated by PZQ treatment rounds and analysed at week 15 i.e. P1 **(C)** Liver Egg burdens are displayed for mice after first (1°), second (2°) and third (3°) infection cycle with 35 *S. mansoni* cercariae after 25 weeks *i.e.* P2. PZQ treatment was administered daily at 400mg/kg by oral gavage twice at the end of each infection cycle of 6-8 weeks. The interrupted line represents the linear prediction of tissue egg burden after repeated infection hypothetically similar in susceptibility as infection 1°. **(D)** Liver egg burden changes in animals undergoing their first (0 to 1°), second (1° to 2°) or third (2° to 3°) cycle of infection intercalated by PZQ treatment rounds at week 25 *i.e.* P2. Data shown are means ± SEM from one of 2 experiments, with 4-7 mice per group in each experiment. NS = p > 0.05; * = p < 0.05; ** = p < 0.01 as determined by Kruskal-Wallis test with correction for multiple comparisons by Dunn’s test **(A, C, D)** or by two-tailed Mann Whitney U test **(B)**.

Collected liver pieces retrieved from animals from each group were processed for egg burden determination as a proxy for susceptibility to infection, similarly to field assessment of infection in humans. Whereas liver egg burdens over cycles of reinfection (0, 1 and 2) did augment significantly early on when animals were relatively younger *i.e.* sacrifice at week 15 (**Figures 2A, B**, p0 vs 2° =0.0037), the liver egg burdens did not significantly increase over cycles of reinfection when assessed at an older age through 25 weeks of follow up *i.e.* P2 at the third round of infection (**Figures 2C**, p 1°vs 2°=0.27; p 2°vs 3°=0.96; p 1°vs 3°>0.99). In fact analyses made at week 25 revealed that liver egg burden changes did not increase over infection cycles, plateauing at increments of around 200 eggs per 20 µg of liver over the first and second cycles of infection-treatment (**Figure 2D**, p 0 to1° vs 1°to2°>0.99). On the third cycle of infection after treatment in P2, we rather noted a significant diminishment of the liver egg burden accumulation to a near zero increment (**Figure 2D**, p 0 to 1° vs 2°to 3°=0.003; p 1°to 2° vs 2°to 3°=0.06) indicating a relative loss of permissivity (to additional egg deposition) by the animal from the third cycle of reinfection (3°) with *S. mansoni*.

These data suggest a reduced susceptibility of mouse to reinfection both over age and over cycles of *S. mansoni* infection interspersed by PZQ treatment.

### Repeated Cycles of *S. mansoni* Infections and PZQ Treatments Viably Induce the Increase of Serum Titres of Schistosoma-Specific Antibodies

**Figure 3:**
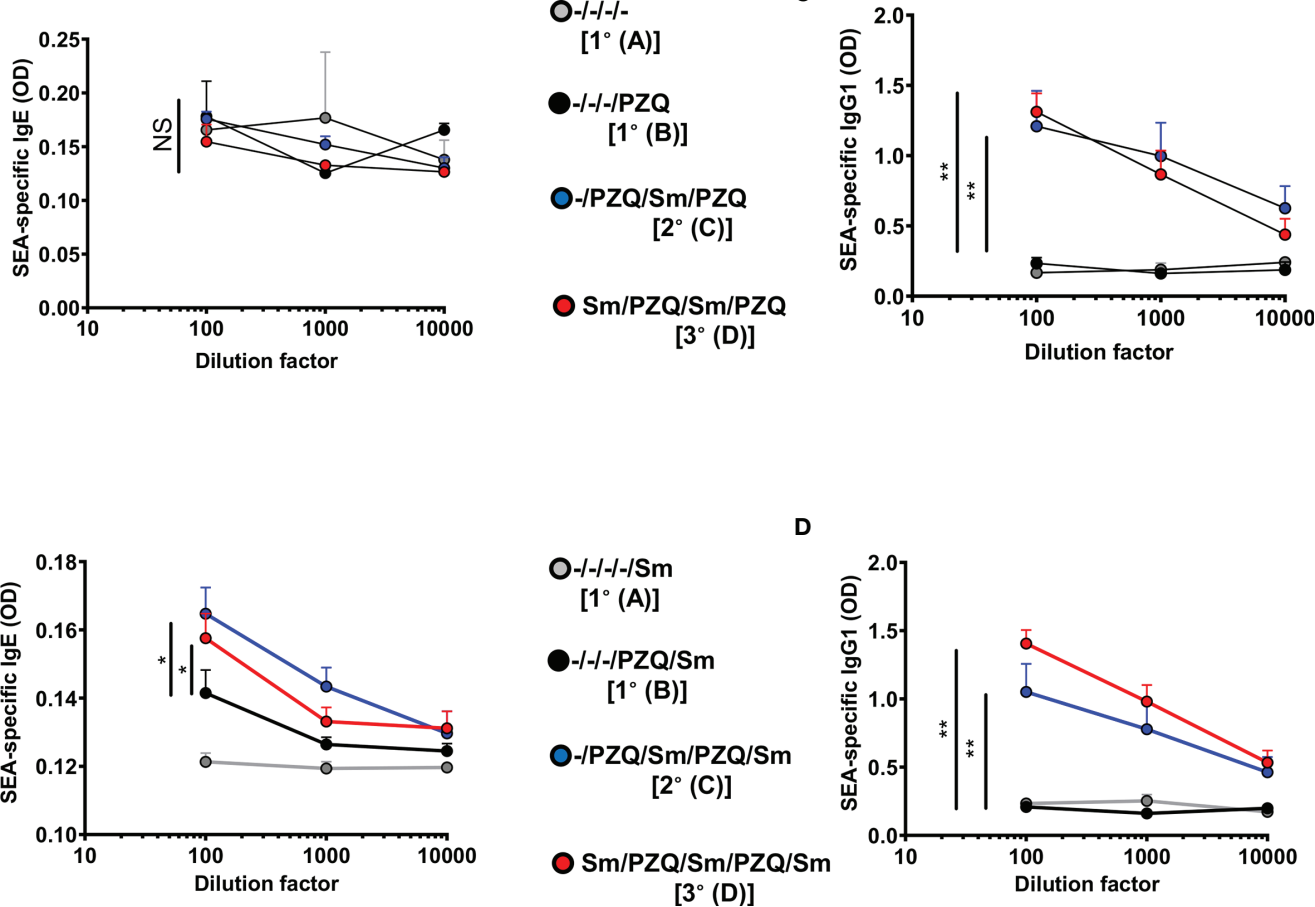
SEA-specific antibodies in serum after repeated cycles of *S. mansoni* infection and PZQ treatments. Outcome measure = Parasite-specific antibody responses in animals. Serum was isolated from animals at the end of the follow-up periods of 15 weeks (P1) and 25 weeks (P2) and parasite-specific antibody responses were measured by ELISA *i.e.* P1 Egg antigen-specific IgE **(A)**, IgG1 **(B)** and P2 Egg antigen specific IgE **(C)** and IgG1 **(D)** are shown. Data shown are means ± SEM from one of 2 experiments, with 4-7 mice per group in each experiment. NS = p > 0.05; * = p < 0.05; ** = p < 0.01 as determined by Kruskal-Wallis test with correction for multiple comparisons by Dunn’s test for the 100 x dilution point.

We further investigated the changes in serum titres of *Schistosoma*-specific antibodies, as a proxy for acquired specific immunity (**Figure 3**). Multiple cycles of infection-treatment in the viable groups included (**Figure 1**) first revealed static levels of parasite-specific IgE (**Figure 3A**) but a rapid increase of parasite-specific IgG1 in mice undergoing one or two cycles of infections when compared to naïve mice (**Figure 3B**) following analyses at week 15 (P1) where mice for a given scheme, say 1° or 2°, are infected at a relatively younger age than animals on the same schemes 1° and 2° in the P2 design. In fact, more prolonged follow up of the animals to ensure three cycles of infections (week 25, P2) revealed a significant increase (p<0.05) of both the serum levels of *Schistosoma*-egg-antigen-specific IgE (**Figure 3C**) and IgG1 (**Figure 3D**), in the groups 2°and 3°, undergoing two or three cycles of infection-treatment respectively, when compared to mice undergoing a single infection cycle (1°). We noted a robust elevation of parasite-specific humoral response following repeated cycles of schistosomiasis infection and chemotherapy, particularly antigen-specific (**Figure 3**) and total (**Figure S4**) IgE levels, in line with the observed reduced susceptibility of repeatedly infected-treated mice to infection when compared to more susceptible counterparts.

Further analyses of serum cytokine levels in these animals revealed elevated levels of IL-4 in mice after two cycles of infection-treatment significantly in the 15 weeks long design (**Figure S5A**) or minimally in the 25 weeks long design (**Figure S5B**). Overall, an observable trend of higher serum levels of IL-4 and TNF-α after two cycles of infection-treatment was apparent and diminished during the third cycle (**Figure S5**). This may suggest a transient elevation of these serum cytokine levels, particularly IL-4, over cycles of infection-treatment to reduction at the third cycle as the host apparently gains more regulation and the ability to oppose reinfection, most likely aided by parasite-specific antibody levels that are gradually elevated (from naïve mice to mice undergoing third cycle of infection-treatment).

### Repeated Cycles of *S. mansoni* Infections and PZQ Treatments and Host Granulomatous and Fibropathological Responses to Trapped Parasite Eggs

We further investigated the effect of cycles of *S. mansoni* infections and chemotherapy with PZQ on the liver periova inflammatory response (**Figure S6**). Fixed liver sections were stained with Hematoxilin-Eosin and the area of the perioval granulomas in the liver were measured by blinded operators (see *Material and Methods*) using the microscope micrometer. Whereas granulomas from mice undergoing 1 or 2 cycles of infection-treatment over a 15 weeks (**Figures S6A, B**) or a 25 weeks (**Figures S6C, D**) did not considerably differ in size (p 1° vs 2° = 0.99), we noted a drastic reduction of granuloma size after three cycles of infection-treatment and over a follow up period of 25 weeks (**Figure S6D**, p 1°A vs 3° <0.0001; p 1°B vs 3° = 0.0002; p 2° vs 3° = 0.0078). This is, in part, suggestive of a diminished cellular responsiveness against trapped eggs in the liver of repeatedly infected and treated animals whatever the cause might be. To further explore this likelihood of reduced cellular responses, liver sections were processed into single cells and immunostained with surface antibodies for the identification of cell types known to participate in the formation of periova granulomas in mouse livers (**Figure S7**). Proportions of liver CD4+ T cells (**Figure S8A**), CD23+ B cells (**Figure S8B**), SiglecF+CD11b+ eosinophils (**Figure S8C**), Lin-T1/ST2+ ILC2 (**Figure S8D**) and F4/80+CD11b+ inflammatory macrophages (**Figure S8E**) were significantly reduced (p<0.05), and consistently so over the third infection & treatment cycle. This indicated a diminished cellular recruitment and/or expansion around trapped *S mansoni* eggs in liver tissue following repeated infection & treatment cycles and/or over extended period of infection-treatment time, similarly to what is observed during prolonged chronic infections (16) when compared to the response in the livers of mice subjected to less repeated but also less prolonged rounds of infection-treatment. Arginase-1 expression during prolonged exposure through infection-treatment cycles significantly increased to achieve significance at the third cycle (**Figure S8F**) when compared to the arginase levels in inflammatory liver macrophages of mice subjected to a single cycle of infection-treatment *i.e.* also over a less prolonged infection duration time. Together, our assessments of the host granulomatous responses in our settings support the reduced cellular inflammatory response that is pathognomonic of prolonged schistosomiasis infection apparent when the chronicity settles, as observed in our animal groups subjected to more repeated infection-treatment cycles.

**Figure 4:**
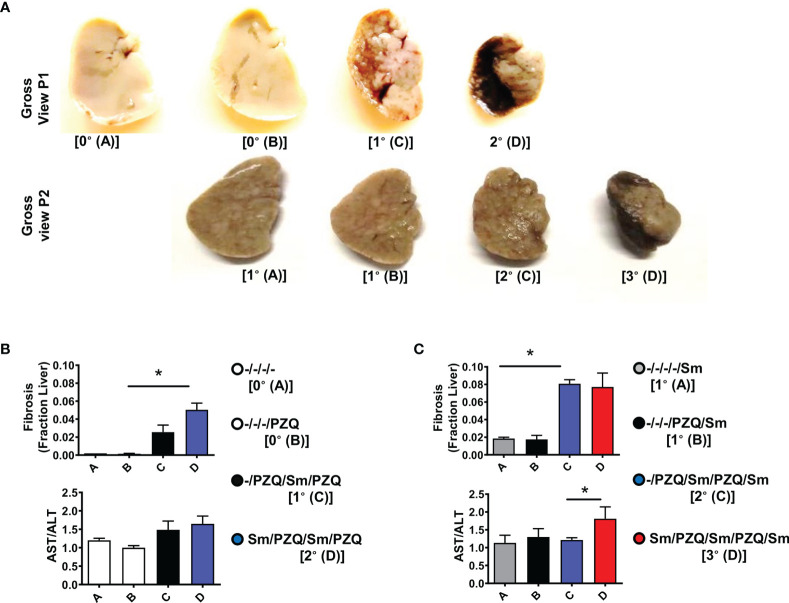
Liver fibropathology after repeated cycles of *S. mansoni* infection and PZQ treatments. Outcome measure = Advancement of liver fibroproliferative processes in animals **(A)** Upper panel shows a gross view of larger liver lobe of mice after differential scheme of infection/PZQ treatments after 15 weeks (P1) and lower panel illustrated the liver lobes after 25 weeks (P2). Note that resolution for both time points differs. For P1, **(B)** upper panel shows Fraction areas of fibrosis (blue deposition of collagen) over the entire lobe scanned are computed as a proxy for the stage of advancement of liver fibrosis and lower panel shows serum AST/ALT ratios computed for mice groups subjected to different schemes of infection/PZQ treatments. For P2, **(C)** upper panel shows Fraction areas of fibrosis (blue deposition of collagen) over the entire lobe scanned are computed as a proxy for the stage of advancement of liver fibrosis and lower panel shows serum AST/ALT ratios computed for mice groups subjected to different schemes of infection/PZQ treatments. Data shown are means ± SEM from one of 2 experiments, with 4-7 mice per group each. * = p < 0.05 as determined Kruskal-Wallis test with correction for multiple comparisons by Dunn’s test. Only significant comparisons between groups are displayed.

Since tissue fibrosis is another pathological hallmark of schistosomiasis (17). We examined the fibropathological profile of mice repeatedly infected with *S. mansoni* and treated with PZQ. Liver lobes showed a clear aggravation of the liver appearance with the progressive appearance of dark, quasi –necrotic areas on the liver over repeated cycles (**Figure 4A**, both after 15 weeks and 25 weeks). Chromotrope Aniline Blue (CAB) staining of liver section revealed a considerable increase of collagen deposits over repeated cycles, as judged by computer-assisted microscopical quantification (**Figures 4B, C**, upper panels). Moreover, serum titres of liver enzymes showed an increased AST/ALT ratio over infection-treatment cycles gradually progressing after 15 weeks in animals undergoing one or two cycles of infection-treatment (**Figure 4B**, Lower panel) to achieve significance in longer term exposure after three infection-treatment cycles (**Figure 4C**, lower panel, p=0.038) indicating overall an increased hepatotoxicity after more prolonged exposure in 3 cycles of infection-treatment.

Tissue cytokines have been reported to be instrumental in the local fibroproliferative response during schistosomiasis (15, 17–19). To understand the mechanistic bases of the aggravated fibroproliferative pathology over infection-treatment cycles thus duration of infection time in our model, we measured the cytokine content of liver homogenates recovered from the tested animals (**Figure S9**). We observed that liver homogenate levels of all tested cytokines (IL-4, IL-13, TGF-β, TNF-α, IL-17, IFN-ɣ and IL-10) were considerably elevated in the livers of animals undergoing three cycles of infections (3°) when compared to less infected-treated animals (1° & 2°) supporting the observed aggravated fibropathology for animals undergoing more cycles of infection-treatment upon a more extended period of exposition (apparent after 25 weeks but not 15 weeks of follow-up). Puzzlingly, tissue cytokine levels in the livers of repeatedly infected animals under P1 design (Upper Panel **Figure 4A** and **Figure S9A**) were not elevated when compared to naïve livers arguing against a key role of the measured tissue cytokines in driving the observed aggravation of liver fibropathology in our setting.

Taken together, nevertheless, our data show an aggravation of fibropathology amid reduced granulomatous response to trapped *S. mansoni* eggs in the livers of potentially more prolonged infections with more infection/treatment cycles, similarly to the known profile of chronic schistosomiasis.

### Impact of *S. mansoni* Infection and PZQ Treatment on Host Immune Responses

**Figure 5:**
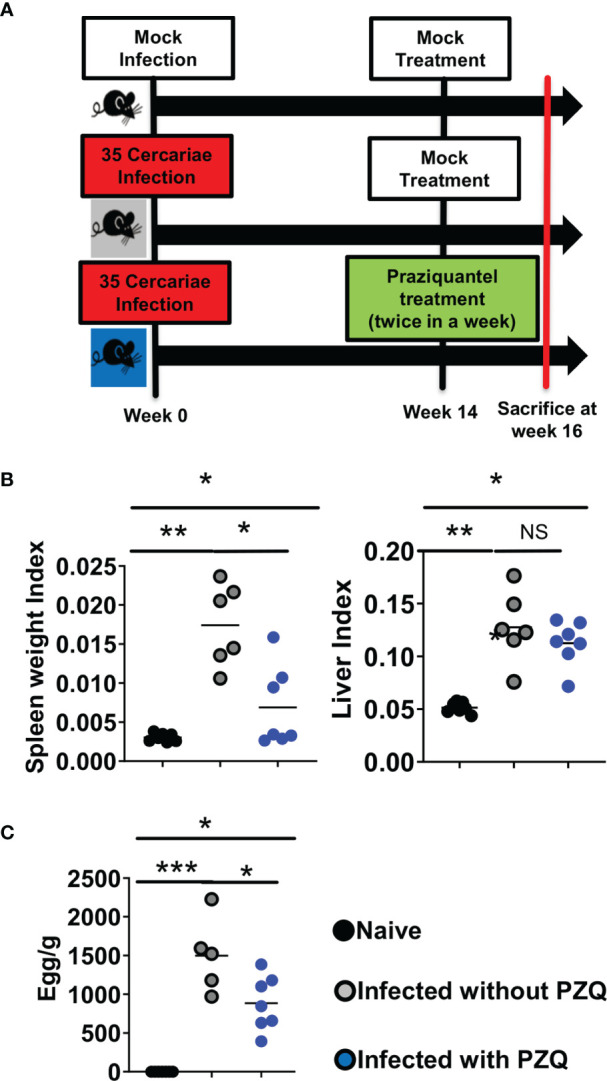
Abridged study design of Infection-PZQ treatment combo. **(A)** study design. Outcome measure = Egg burdens and rate of associated organomegaly in animals. C57BL/6 (>8 weeks old) mice were infected percutaneously with 35 *S. mansoni* cercariae (or exposed to cercariae water for the naïve group) and treated 14 weeks later either with racemic PZQ (or carrier solution for the infected and untreated group) by administration at 400mg/kg twice within a week by oral gavage. Two weeks following the end of anti-parasitic treatment with PZQ, mice were sacrificed and, specifically, biopsies of spleen, liver and blood were done for laboratory analyses of egg burdens, Antibody ELISA, Cytokine ELISA, Histology and Flow cytometry. **(B)** Organ weights (liver and spleen indexes as ratio of organ to body weights). **(C)** Liver egg burden. Experiments were conducted in two independent runs with 5-7 mice per group (Experiment 1: naïve group = 7 mice; infected group = 6 mice; infected and treated group = 7 mice; Experiment 2: naïve group=5 mice; infected group = 5 mice; infected and treated group = 7 mice). A total of 37 mice were used for this scheme. Data are expressed as mean ± SEM; NS = p > 0.05; *= p < 0.05; **= p < 0.01; ***=, p < 0.001 as determined by Kruskal-Wallis test with correction for multiple comparisons by Dunn’s test.

To assess the immunological impact of PZQ treatment (by oral gavage twice during week 6 post infection for deparasitization) on animals infected with the optimized inoculum of *S. mansoni* cercariae (35 cercariae), their tissue, cytokine and IgE responses were measured following an abridged experimental scheme of infection and treatment (**Figure 5A**). On their tissue responses, we first confirmed a significant induction of hepato-splenomegaly by *S. mansoni* infection (**Figure 5B**) that paralleled a significant ability of PZQ treatment to reduce liver egg burden as a proxy for therapeutic efficiency of PZQ treatment in our setting (**Figure 5C**).

**Figure 6:**
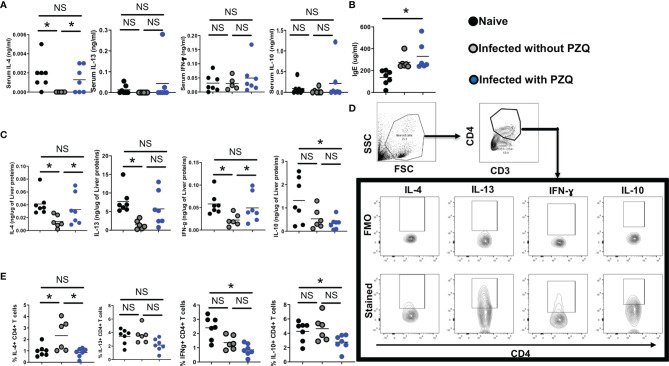
Impact of infection and Infection-PZQ treatment combo on host immune response. Outcome measure = Change in immune markers in animals. **(A)** Serum total cytokine levels **(B)** and total IgE levels were determined by ELISA in ng/ml and ug/ml respectively. **(C)** Liver lobes collected from animals treated according to the differential scheme and sacrificed at week 16 were homogenized and the production of cytokines (IL-4, IL13, IFN-g and IL-10) was determined as a function of the protein amount of liver tissue probed. Liver lobes were processed for FACS analysis and the gating of cells and cytokine-producing CD4+ T cells defined as shown in **(D)**. **(E)** Summary frequencies of cytokine-producing hepatic CD4+ T cells. Experiments were conducted in two independent runs with 5-7 mice per group. Data are expressed as mean ± SEM; NS = p > 0.05; *= p < 0.05 as determined by Kruskal-Wallis test with correction for multiple comparisons by Dunn’s test.

Serum canonical cytokines (IL-4 and IL-13 for Th2, IFN-ɣ for Th1 and IL-10 for regulation) were assessed (**Figure 6A**). We noted a drastic reduction of serum cytokine levels caused by *S. mansoni* infection that was partially restored by PZQ treatment (**Figure 6A**) arguing for a normalization of the immune dysregulation of the infection by PZQ treatment. Conversely, we noted a strong IgE induction by the parasite in the host serum that was sustained even after PZQ treatment (**Figure 6B**) in infected then treated mice, supporting an advantageous immune disposition of infected-treated mice when compared to naïve mice, given the strongly suggested protective role of serum IgE against schistosomiasis reinfection (20, 21). In the tissue (liver, **Figure 6C**), infection similarly to what was observed in the sera, led to a significant reduction of cytokine levels which were normalized by PZQ treatment supporting a non-protective disposition of the host tissue to the parasite trapped eggs even after undergoing an infection and a treatment cycle. Such an observation argues in support of the continued cytokine-supported part of the fibropathological response of infected-treated mice to newly deposited eggs over subsequent cycles of infection and treatment. Cytokine production by Liver CD4 T cells (**Figure 6D**) remained relatively low following infection and treatment (**Figure 6E**) providing an additional support to the reduced granulomatous response observed after repeated cycles of infection-treatment. Overall, it appears that PZQ treatment after infection normalizes serum and tissue responses to ensure a continued immunopathological response to newly deposited parasite eggs while reducing the CD4+ T cell specific tissue responses instrumental in the host granulomatous responses. Unprecedentedly, our data reveal the maintenance of a heightened – and potentially protective - IgE response in the serum of infected then treated animals when compared to naïve animals that might predispose the infected-treated host to a more protected response to reinfection.

## Discussion

Reports over the last three decades have constantly emerged on the non-conventional additional benefits of repeated PZQ treatment in endemic areas (22–27) and in experimental models (19). These warranted the WHO recommendation of intensified treatment rounds in endemic persistent areas to foster the 2030 Neglected Tropical Diseases (NTD) road map goal of attempting schistosomiasis elimination as a public health problem (3). A clear definition of the breadth of consequences of more frequent cycles of infection followed by treatment in a controlled experimental model would therefore be highly informative.

Using the mouse model of *S. mansoni* chronic infections, similarly to natural infections in the field (15), we investigated the susceptibility to reinfection after exposing mice to an increasing number of infections followed by a robust scheme of effective PZQ treatment cycles. Even after two infection-treatment cycles, mice were still found to be susceptible to infection to a low dose of 35 cercariae of *S. mansoni*, indicating the lasting susceptibility of the host to contracting new schistosomiasis infection after re-exposure, even after multiple cycles of infection 2-3 weeks after two treatments with PZQ within a week [similarly to the cycles of infection and treatment regimens affecting individuals in the field (14)]. This is consistent with previous observations in mice (12), in baboons (18) and in children (28) infected with *S. mansoni* or *S. haematobium*. These observations across species suggest an apparent inability of the host to acquire sterile immunity (complete protection) against schistosomiasis reinfection even after previous exposures or the ability of the worm to circumvent the host immune system (29). In some instances of parasitic infections, the levels of IgE and eosinophils permits to control the disease in some patients when levels are high, but as soon as these levels drop, the risk of reinfection might come back to the baseline (30, 31). Notably in our study, retrieved livers showed a reduced trend of egg accumulation after any additional infection-treatment cycle indicating the reduced success of the parasite larvae infestation capacity and/or of the parasite egg-generating ability after the re- exposition of infected-treated (sensitized) animals to *S. mansoni* cercariae. This is consistent with a previous seminal study (26) that demonstrated infection-treatment driven protective immunization in the context of schistosomiasis. Such a partial but accelerated acquisition of protection against schistosomiasis has now been increasingly reported by several groups and in several models (26, 27, 31–34). Some of these previous studies further noted a rapid increase of parasite-specific humoral responses in partially protected hosts (26, 27, 33–35) which closely resemble our own observation of elevated SEA-specific IgE particularly in the serum of mice over infection-treatment cycles. Eventual differences in age or gender as previously reported in clinical studies (26) were minimized in the presently controlled study by age-and-gender-matching of the different groups of animals. Nevertheless, the prolonged exposure of animals undergoing more rounds of infection – treatment introduces a confounder that is longer exposure time to worms in groups undergoing more rounds of infection-treatment, where the administered treatment is consistent with the clinical administration of PZQ to individuals in the field during MDA campaigns *i.e.* full cure is rarely observed (1, 5, 9, 13, 20, 21, 27, 28) and cure rates oscillate around ~76% (5).

To correct for the high likelihood of non-sterile clearance of the infection by the PZQ treatment administered in our infection-treatment cycles, a single infection-treatment scheme was further dissected in this study revealing a similarly heightened ability of treated mice to retain protective IgE levels. Therefore, this observation argues in favour of our conclusion of infection-treatment driven acquisition of protective immunity. As such, the rapid increase of parasite-specific antibodies might most likely come as a result of the accelerated rounds of parasite destruction by the repeated cycles of treatment and the consequently more abundant release and exposure of antigens to the immune system of these animals.

We also noted a significant reduction in the average periova granuloma size in animals with higher numbers of infection-treatment cycles; An observation that indicates an anti-granulomatous inflammation potential of repeated infection-treatment cycles in the context of the regular administration of PZQ. This is in line with previous studies in baboons (19) and mice (36) where a striking reduction of egg-driven granulomatous inflammation paralleled repeated cycles of schistosomiasis infection. In our setting however, the reduced granulomatous response might also be a direct result of the prolonged duration of infection in the more repeatedly infected-treated groups where drug administration does not necessarily equate a 100% cure rate [median worm burden reduction = 97% (84 ± 100)] (37). As such, persistent infection could potentially develop to chronicity and support a reduced granulomatous response to trapped eggs as previously reported (38).

Mechanistically, reduction of cell frequencies in liver tissue of more infected-treated groups of mice might explain the diminished granulomatous inflammation (31, 39, 40). We postulate here that the repeated/prolonged exposure to the parasite antigen might have rendered the host cellular machinery tolerant to further parasite antigen and possibly elicited the preferential expansion of regulatory arms of the immune response in the event of any subsequent challenge. In fact, the augmentation of the liver expression of the regulatory cytokine IL-10 argues in favour of such an underlying regulatory immune network paralleling prolonged/repeated exposure to infection in our setting. Notably, however, the continued augmentation of most cytokine released here could simply be due to the chronic nature of the infection in animals over cycle of infection/treatment. The general destruction of the liver parenchyma could be resulting from this continued inflammatory solicitation. In fact, we also noted an augmentation of collagen deposition over cycles of infection-treatment indicating a continued increment of liver fibrosis caused by repetitive cycles of infections. This is in agreement with a previous report by Farah and collaborators (18) that reported on the ability of repeated exposure to *Schistosoma mansoni* to rapidly drive periportal fibrosis in baboons.

We also noted a significant elevation of all cytokine tested (*i.e.* IL-4, IL-13, TGF-β, TNF-α, IL-17 and IFN-ɣ) in liver homogenates, consistent with a pro-fibrotic profile (15, 17). However, this parallels a loss of parenchyma and reduction of inflammatory immune cells within the liver either suggesting a non-immune source of all the reported cytokines (41) and/or the possibility of the reported heightened inflammatory cytokine production occurring prior to (and possibly causing) the cell death, loss of parenchyma and aggravated fibrosis observed. In fact, the immediate response to necroinflammation and cellular death has been reported to be the extensive deposition of extracellular matrix (ECM) by activation of Hepatic stellate cells (HSCs) (42) supporting the latter hypothesis. Whatever the case, it stands here to reason to assume that repeated cycles of infection-treatment elicited, a quasi-general cytokine release in the affected liver and an aggravated cytokine/necrosis-mediated fibroproliferative response as a result of the hepatotoxic potential of the parasite eggs. This in turn actively depleted the liver parenchyma and limited the host ability to mount a robust cell-dependent granulomatous response against the trapped eggs. In fact, our abridged infection-treatment scheme further revealed a diminished CD4 T cell cellular responsiveness in the livers of infected-treated animals when compared to infected or naïve animals arguing in favour of a gradual cellular hyporesponsiveness following cycles of infection-treatment. Moreover, the observation in this abridged scheme of the unaltered tissue cytokine levels in infected-treated animals does not argue against the likely aggravation of the tissue cytokine response following cycles of infection-treatment-reinfection en route to the severe fibropathological diseases observed after multiple rounds of infection treatment.

Provided the implications of the present findings are translatable to the community-performed mass drug administration (MDA) with PZQ interspersed by reinfections, the study suggests that the host susceptibility to reinfection appears to be a more quantitative than qualitative process since repeated treatment with PZQ in mice is active not only in directly treating ongoing infections but also limiting the relative susceptibility to subsequent infections. Consequently, our findings in a preclinical mouse model warrant further investigation in the field with high transmission rates to confirm whether MDA of PZQ would foster the host ability to mount protective immune responses against reinfection and as such gradually protect against reinfection teasing out the specific role of cellular and humoral responses in this process. It would therefore also be needed here to assess whether repeated MDA with PZQ amid continuous infection-reinfections do not appear to halt the morbid consequence of aggravated liver fibrosis as observed in our preclinical report. Certainly, praziquantel treatment of naïve hosts could have effects of its own on these cellular and humoral responses and should also be further investigated. In this undertaking where we assessed the combined action of praziquantel and infection, however, we do logically observe a rapid restoration of the tissular immune responsiveness in these animals that was first lost as a result of a quasi-lethal chronic infection (>14 weeks), supporting our claim of a response better poised to adopt an anti-schistosomiasis profile following praziquantel treatment of already infected hosts.

In summary, within the tractable mouse model of hepatosplenic schistosomiasis, repeated infection-treatment cycles led to a relatively reduced susceptibility of the animals to reinfection, as judged by the reduced rate of egg accumulation in liver tissues, over time through cycles of infection-treatment.

## Data Availability Statement

The original contributions presented in the study are included in the article/**Supplementary Material**. Further inquiries can be directed to the corresponding authors.

## Ethics Statement 

The animal study was reviewed and approved by University of Cape Town Animal Ethics Committee.

## Author Contributions

Conceptualization: JKN, FB, and TS. Data curation and Data Analysis: JKN, TM, PM, NA, LH, FM, FB, and TS. Formal analysis: JKN, TM, PM, NA, FM, FB, and TS. Funding acquisition: TS and FB. Investigation: TM, PM, JKN, NA, and FM. Methodology: JKN, TM, NA, PM, TS, and FB. Project administration: JKN, TS, FB. Resources: TS and FB. Supervision: JKN, TS, and FB. Writing – original draft: JKN and TS. Writing – review & editing: JKN, PM, TM, NA, FM, TS, and FB. All authors contributed to the article and approved the submitted version.

## Funding

This work was funded by Merck KGaA, Darmstadt, Germany with support from CIDRI-Africa (grant No 203135/Z/16/Z).

## Conflict of Interest

TS is an employee of Ares Trading SA, an affiliate of Merck KGaA, Darmstadt, Germany. JKN is a founding member of JRJ Health, a health-promoting association based in Cameroon.

The remaining authors declare that the research was conducted in the absence of any commercial or financial relationships that could be construed as a potential conflict of interest.

The authors declare that this study received funding from company Merck KGaA. The funder was involved in the conceptualization, design, analysis of collected data, decision to publish, and preparation of the manuscript.

## Publisher’s Note

All claims expressed in this article are solely those of the authors and do not necessarily represent those of their affiliated organizations, or those of the publisher, the editors and the reviewers. Any product that may be evaluated in this article, or claim that may be made by its manufacturer, is not guaranteed or endorsed by the publisher.
