# Supplementary material for: Praziquantel Treatment of Schistosoma mansoni Infected Mice Renders Them Less Susceptible to Reinfection
Source: Front Immunol. 2021 Dec 10;12:748387. doi: 10.3389/fimmu.2021.748387 (PMC8703194; doi:10.3389/fimmu.2021.748387)
Supplement: Supplementary file 1 [file DataSheet_1.docx]

**Praziquantel treatment of *Schistosoma mansoni* infected mice renders them less susceptible to reinfection**

Justin Komguep Nono^1,2,3¶^, Thabo Mpotje^1,2¶^, Paballo Mosala^1,2^ Nada Abdel Aziz^1,2,4^, Fungai Musaigwa^1,2^, Lerato Hlaka^1,2^, Thomas Spangenberg^5*^, Frank Brombacher^1,2,6*^

**SUPPLEMENTARY FIGURES AND LEGENDS**

**
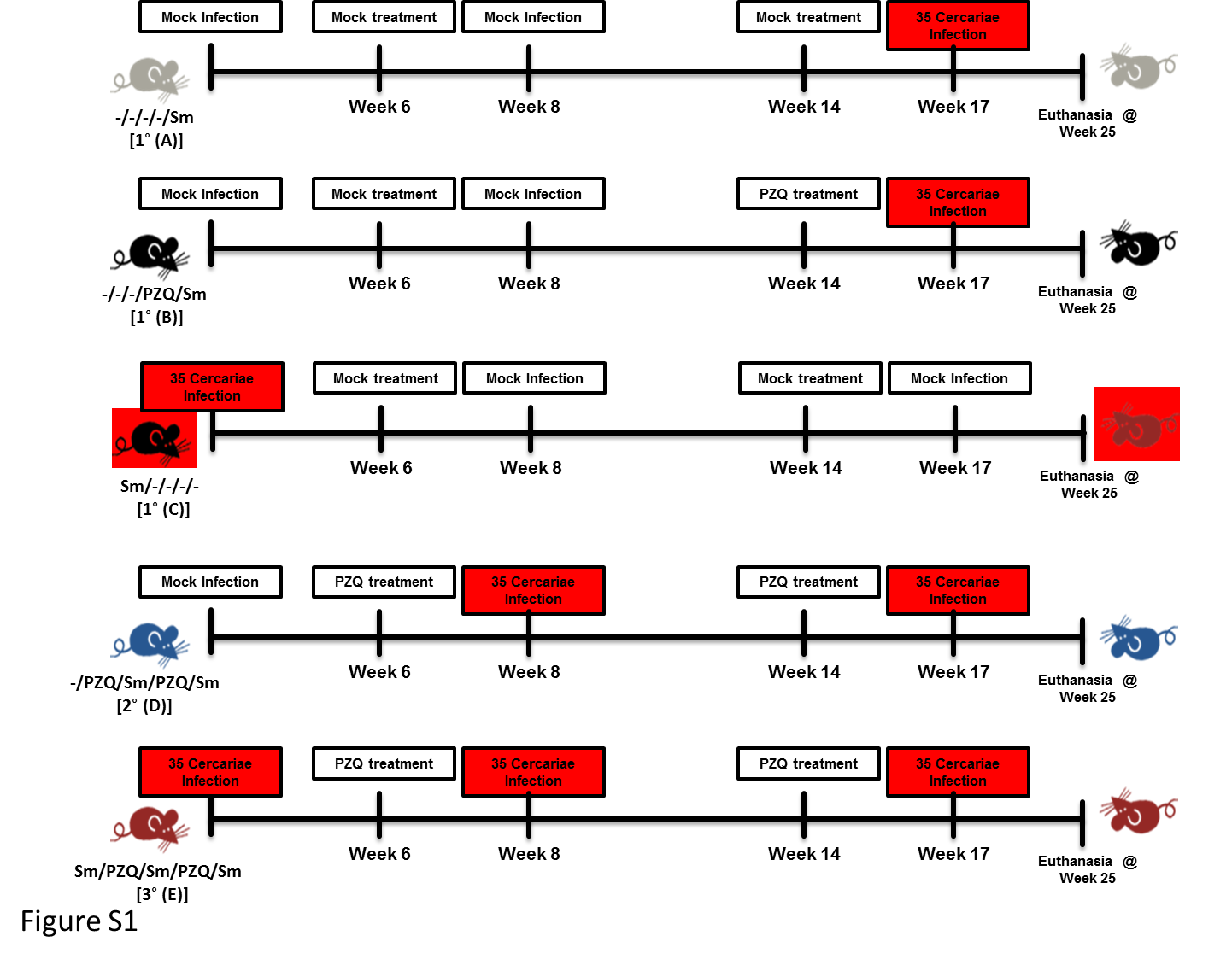
**

**Figure S1: Theoretical study design.** Following Following experimental infection of mice with S. mansoni cercariae and anti-parasitic treatment with racemic Praziquantel (PZQ), the susceptibility to reinfection with S. mansoni and schistosomiasis-driven pathology will be assessed. Briefly, C57BL/6 mice (>8 weeks old) are to be separated into five experimental groups A, B, C, D and E i.e. A for an overall total of 1 late infection and no PZQ treatment i.e. -/-/-/-/Sm, B with a single pretreatment with PZQ and an overall total of 1 infection i.e. -/-/-/PZQ/Sm ; C for a single infection early and no pretreatment and no further infections until the end of the monitoring i.e. Sm/-/-/-/- , D for a pretreatment with PZQ, an overall total of two infections intercalated by another treatment with PZQ i.e. -/PZQ/Sm/PZQ/Sm; D for an overall total of three infections intercalated by two PZQ treatments i.e. Sm/PZQ/Sm/PZQ/Sm, as indicated on the illustration. Practically, mice are to be infected percutaneously with 35 S. mansoni cercariae (or cercariae water as Mock) and treated 6 weeks later with racemic PZQ (or carrier solution) by two administrations at 400mg/kg by oral gavage within a week. One week following the end of anti-parasitic treatment with PZQ, mice are to be reinfected percutaneously with 35 S. mansoni cercariae (or cercariae water as Mock) i.e. at week 8. Animals will then be treated with racemic PZQ (or carrier solution) 6 weeks later i.e. at week 14 by oral administration of PZQ at 400mg/kg by oral gavage twice within a week time. Two weeks after the end of this second round of anti-parasitic treatment i.e. at week 17, all mice, including controls from group A and B, are to be reinfected percutaneously, with 35 S. mansoni cercariae. Animals were then sacrificed 8 weeks later i.e. at week 25 and markers of infection and pathology will be assessed.

**
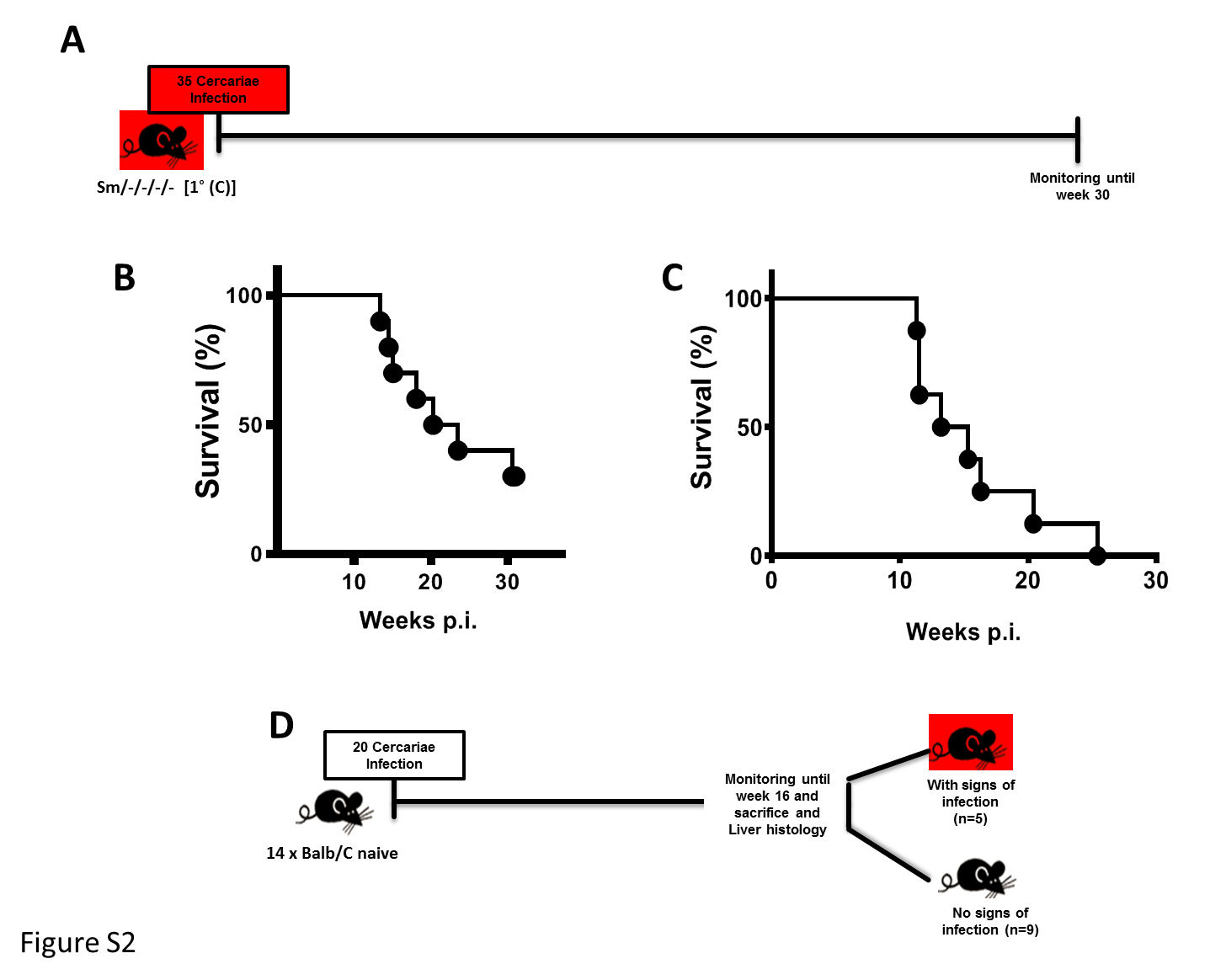
**

**Figure S2: Assessment of mice survival during long term infection without treatment in our settings and infectivity of very low dose infection (20 cercariae) in our settings. Outcome measure = Animal survival. A.** The prospective group C of our theoretical design i.e. C57BL/6 or 129SV mice (>8 weeks old) following experimental infection of mice with *S. mansoni* cercariae (low dose of 35 per animal) were monitored for up to 30 weeks to assess the ability of these mice to survive throughout the implementation of our theoretical experimental design (figure S1). B. Survival curve for C57BL/6 mice infected with *S. mansoni* cercariae (low dose of 35 per animal), n=9 . C. Survival curve for 129SV mice with *S. mansoni* cercariae (low dose of 35 per animal), n=8. D. Infectivity with very low dose of *S. mansoni* cercariae (20 cercariae per animal) in the more resistant C57BL/6 mice after 16 weeks assessed by liver histology, H&E staining.


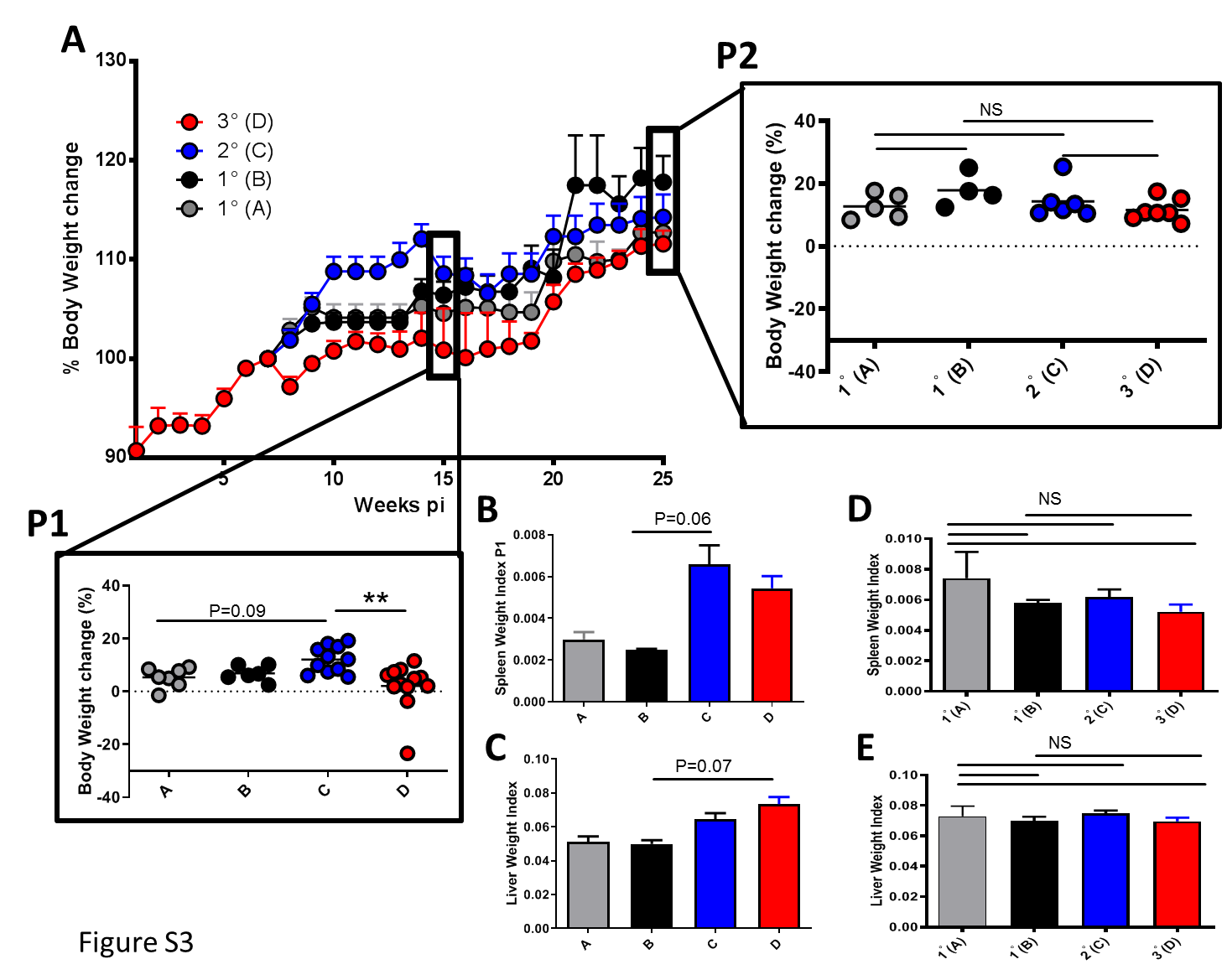


**Figure S3: Body and organ weights. Outcome measure = body and organ weight changes. (A)** Body weights, Changes are represented for each individual animal as percentage of change to the weight recorded at week 7 of the monitoring where all groups presented with similar progression. **(B)** spleen weight indexes at week 15 i.e. P1; **(C)** liver weight indexes at week 15 i.e. P1. **(D)** spleen weight indexes at week 25 i.e. P2; **(E)** liver weight indexes at week 25 i.e. P2. Organ indexes for a given animal represent the percentage of the organ over the total body weight. Data shown are means ± SEM from one of 2 experiments, with 4-11 mice per group in each experiment. NS = p > 0.05; * = p < 0.05; ** = p < 0.01; *** =, p < 0.001; **** = p < 0.0001 as determined by Kruskal-Wallis test with correction for multiple comparisons by Dunn’s test. Non displayed statistical comparisons are not significant.

.


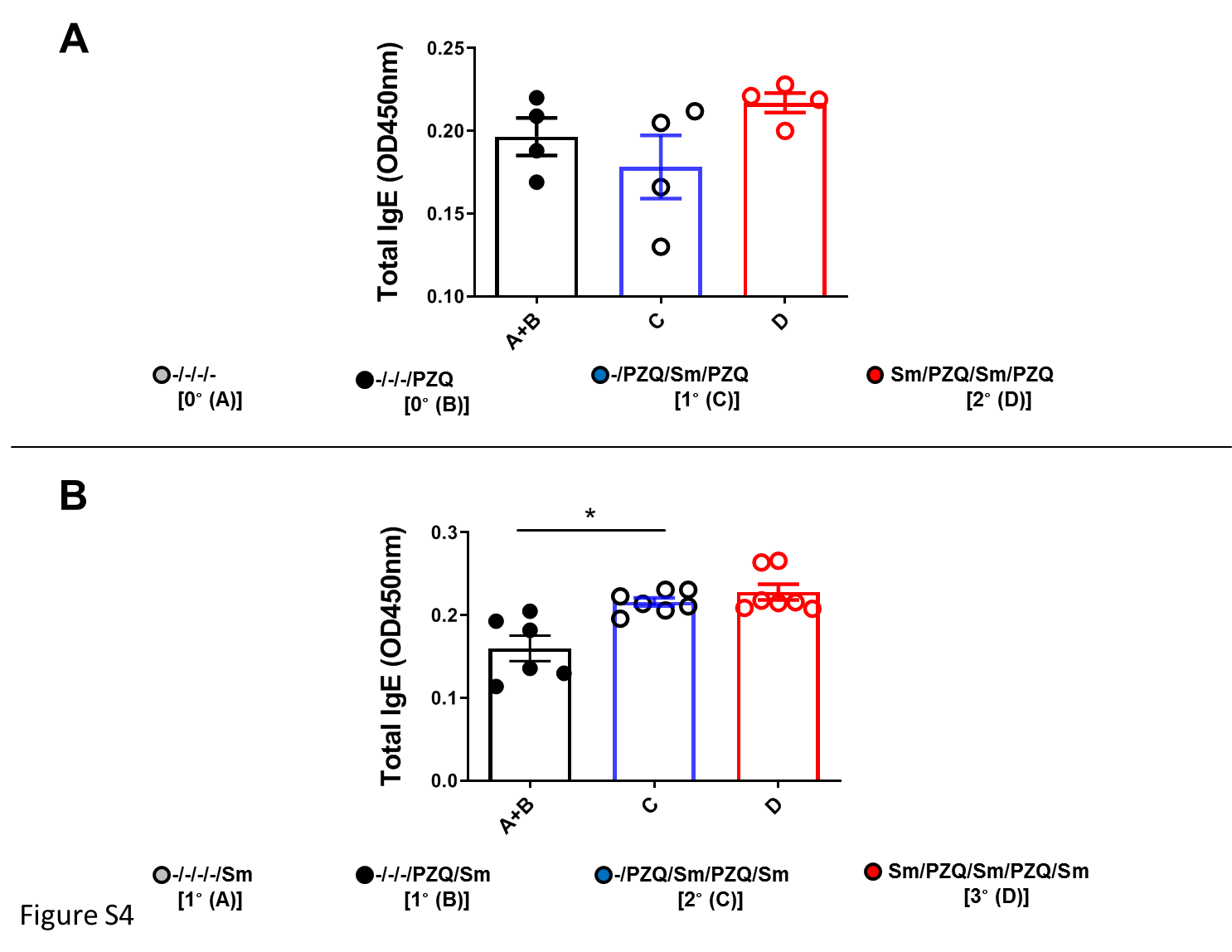


**Figure S4: Serum total IgE levels after repeated cycles of *S. mansoni* infection and PZQ treatments.** Serum was isolated from animals at the end of the follow-up periods of 15 weeks (P1, **(A)**) and 25 weeks (P2, (**B)**) and total IgE levels were determined by ELISA as per the OD450nm. Data shown are means ± SEM from one of 2 experiments, with 4-7 mice per group in each experiment. NS = p > 0.05; * = p < 0.05; ** = p < 0.01; *** =, p < 0.001; **** = p < 0.0001 as determined by Kruskal-Wallis test with correction for multiple comparisons by Dunn’s test.

**
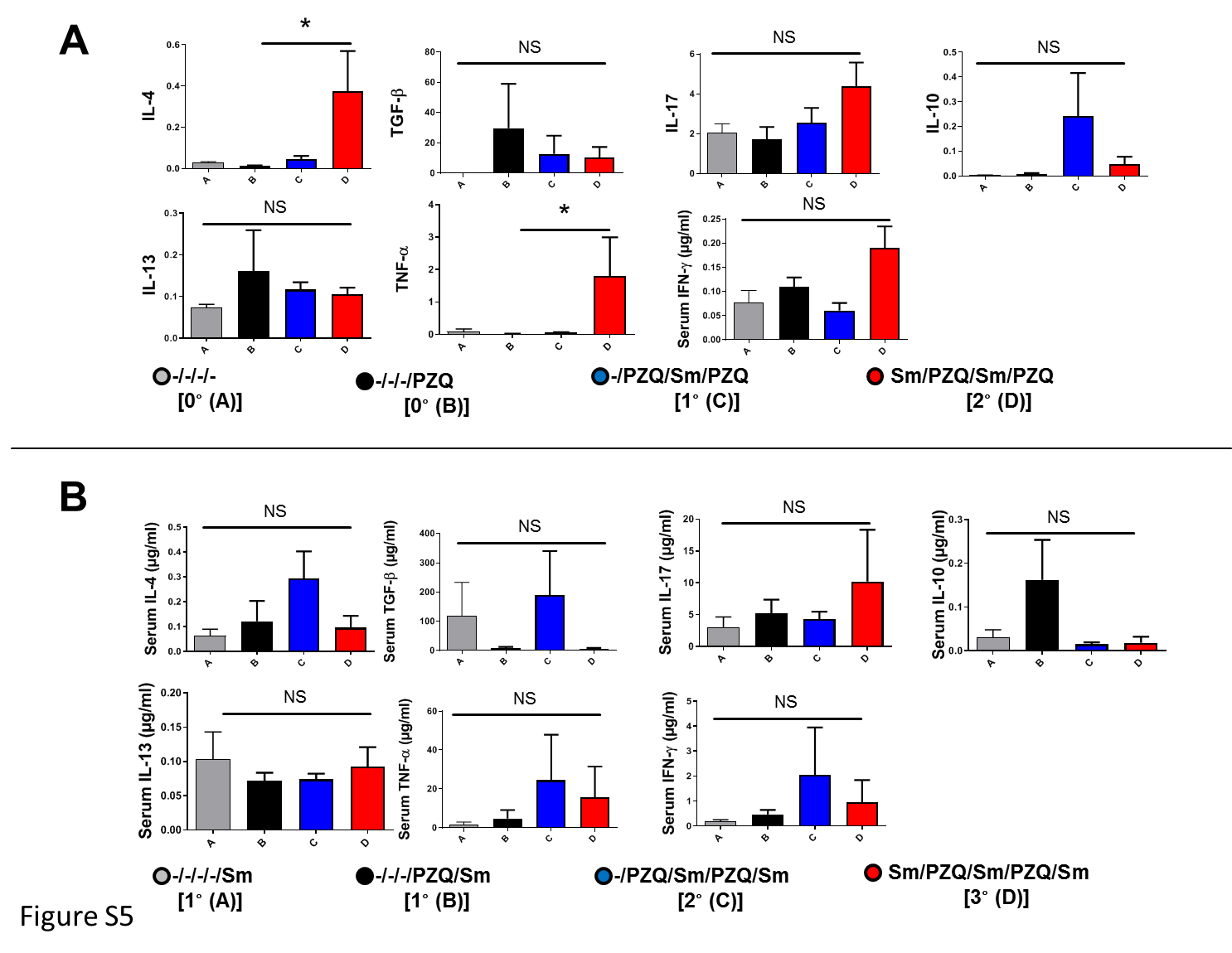
Figure S5: Serum cytokine levels after repeated cycles of *S. mansoni* infection and PZQ treatments. Outcome measure = Change in immune markers in animals.** Serum was isolated from animals at the end of the follow-up periods of 15 weeks (P1, **(A)**) and 25 weeks (P2, (**B)**) and total cytokine levels were determined by ELISA in ug/ml. Data shown are means ± SEM from one of 2 experiments, with 4-7 mice per group in each experiment. NS = p > 0.05; * = p < 0.05; ** = p < 0.01; *** =, p < 0.001; **** = p < 0.0001 as determined by Kruskal-Wallis test with correction for multiple comparisons by Dunn’s test for the 100 x dilution point.

**
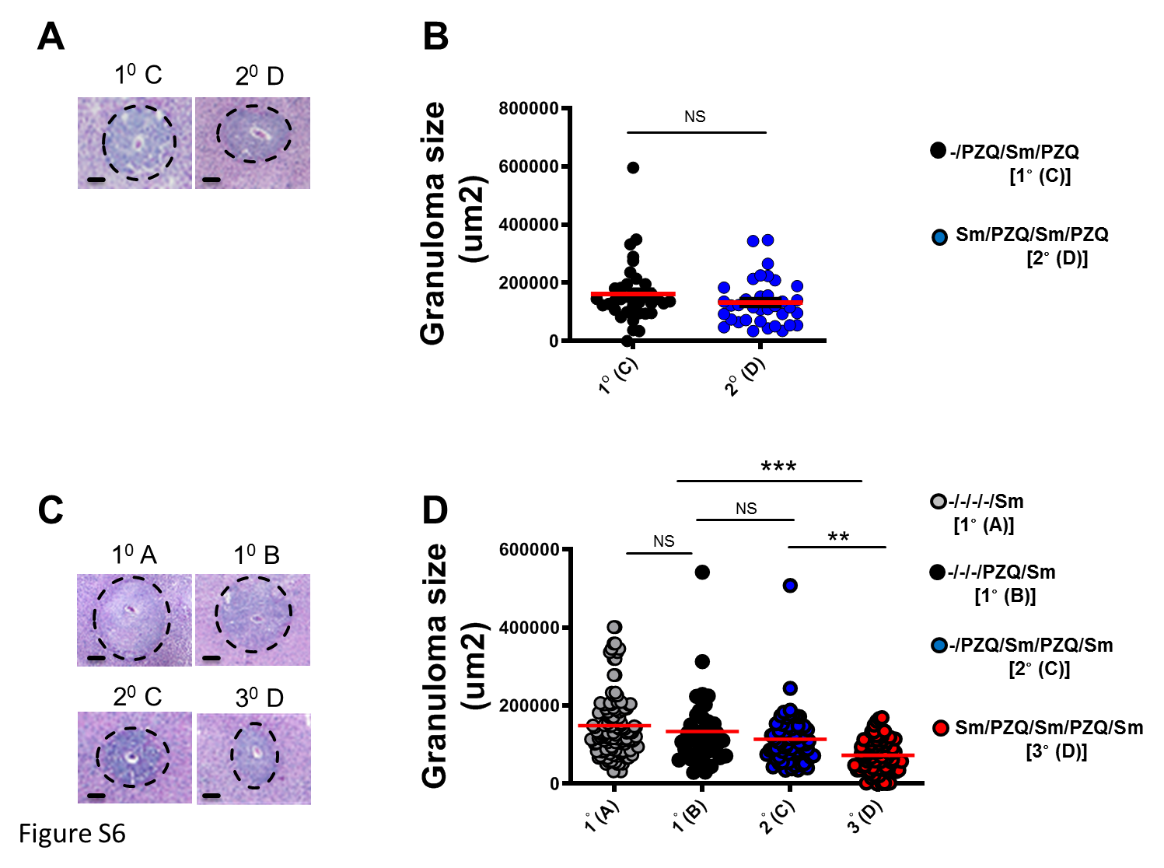
**

**Figure S6: Periova liver granuloma sizes after repeated cycles of *S. mansoni* infection and PZQ treatments. Outcome measure = Parasite-driven tissue pathology in animals.** Livers were excised from animals at the end of the follow-up of 25 weeks. , fixed in formalin, embedded in paraffin and stained with Haematoxilin and Eosin for histological examination by microscopy and Area sizes of egg-surrounding granuloma were computed. Representative granulomas from liver sections of animals sacrificed at week 15 (A for P1) or week 25 (C for P2) are shown and the granuloma areas measured by microscopy are also computed (B for P1 and D for P2). Scale bars in A and C represents 100μm. Data shown are one of 2 experiments, with 4-7 mice per group and 5-10 granuloma analysed per animal liver lobe. Horizontal red lines in B and D represent the means. NS = p > 0.05; * = p < 0.05; ** = p < 0.01; *** =, p < 0.001; **** = p < 0.0001 as determined by two-tailed Mann Whitney U test (B) or by Kruskal-Wallis test with correction for multiple comparisons by Dunn’s test (D).

**
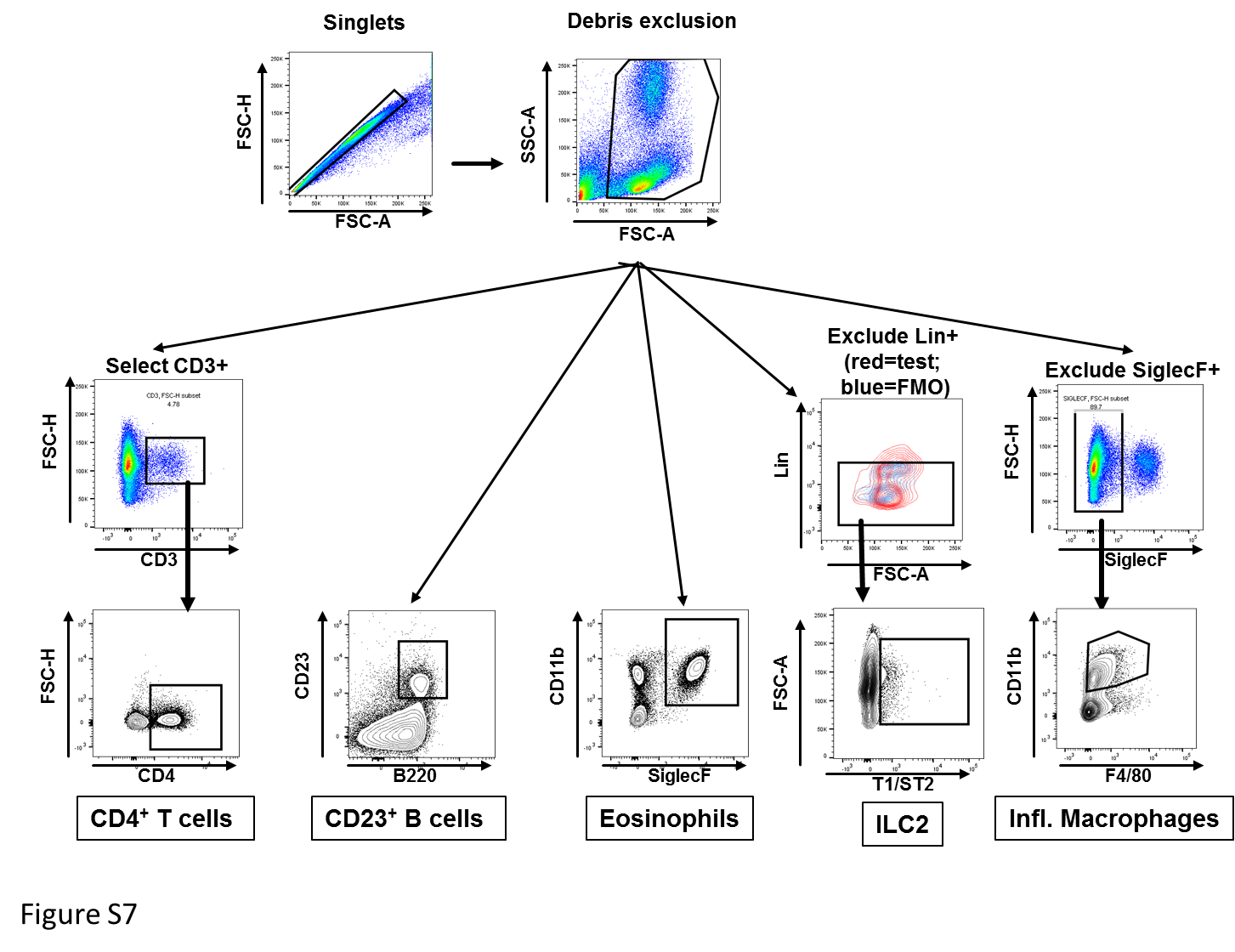
**

**Figure S7: Gating strategy.**  Pipeline to identify liver CD4+ T cells, CD23+ B cells, Eosinophils, ILC2 and Inflammatory macrophages.

**
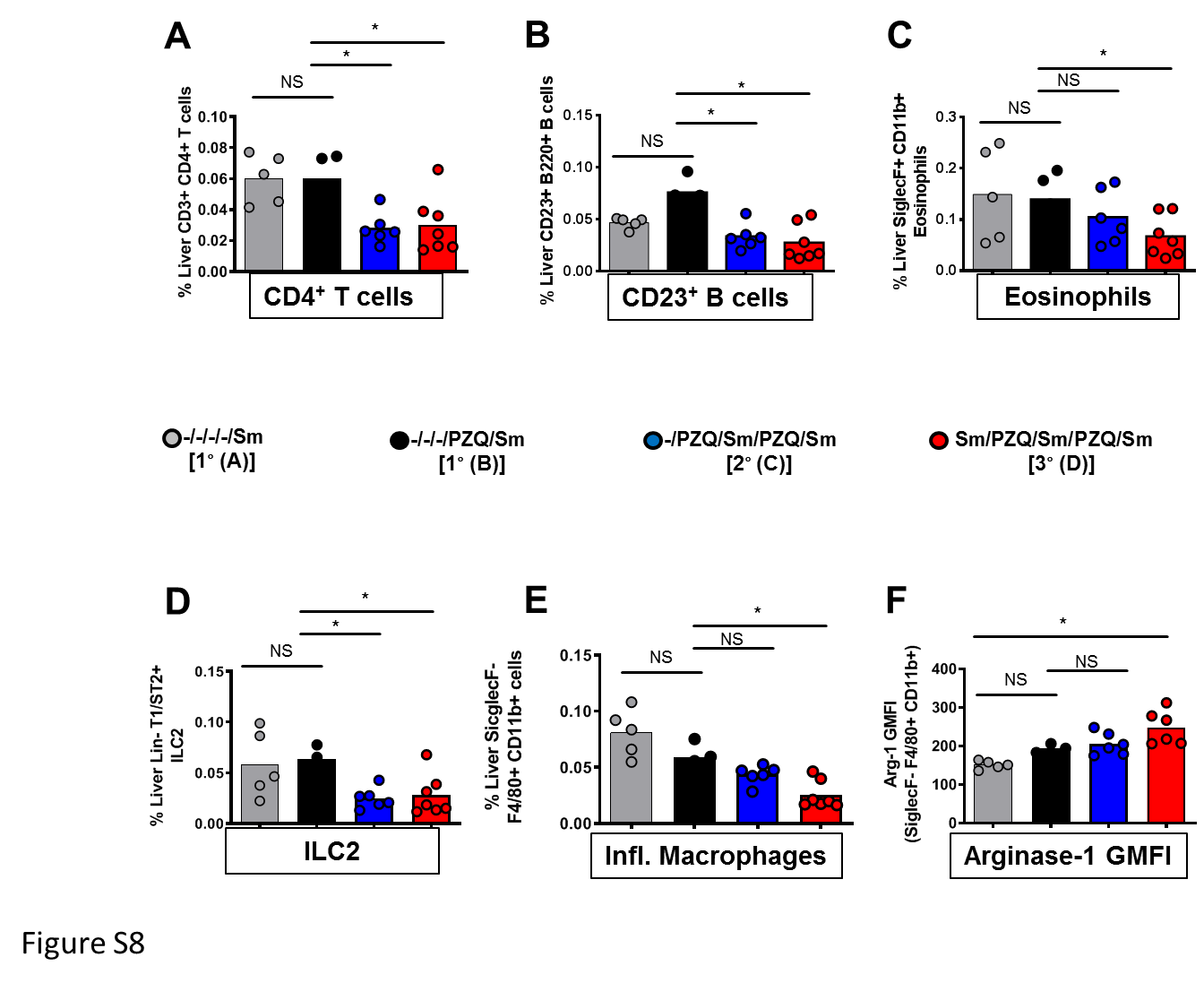
**

**Figure S8: Frequencies of periova liver cells after repeated cycles of *S. mansoni* infection and PZQ treatments. Outcome measure = Change in immune markers in animals.** Total liver cells were separated by flow cytometry for the identification of **(A)** CD3+CD4+ T cells, **(B)** CD23+ B cells,  **(C)** eosinophils, **(D)** ILC2, **(E)** Inflammatory macrophages and **(F)** the propensity of arginase-1 production by inflammatory macrophages as judged by GMFI. Data shown are means from one of 2 experiments, with 4-7 mice per group for each experiment with 2 liver lobes jointly analysed per animal. NS = p > 0.05; * = p < 0.05; ** = p < 0.01; *** =, p < 0.001; **** = p < 0.0001 as determined by Kruskal-Wallis test with correction for multiple comparisons by Dunn’s test.

**
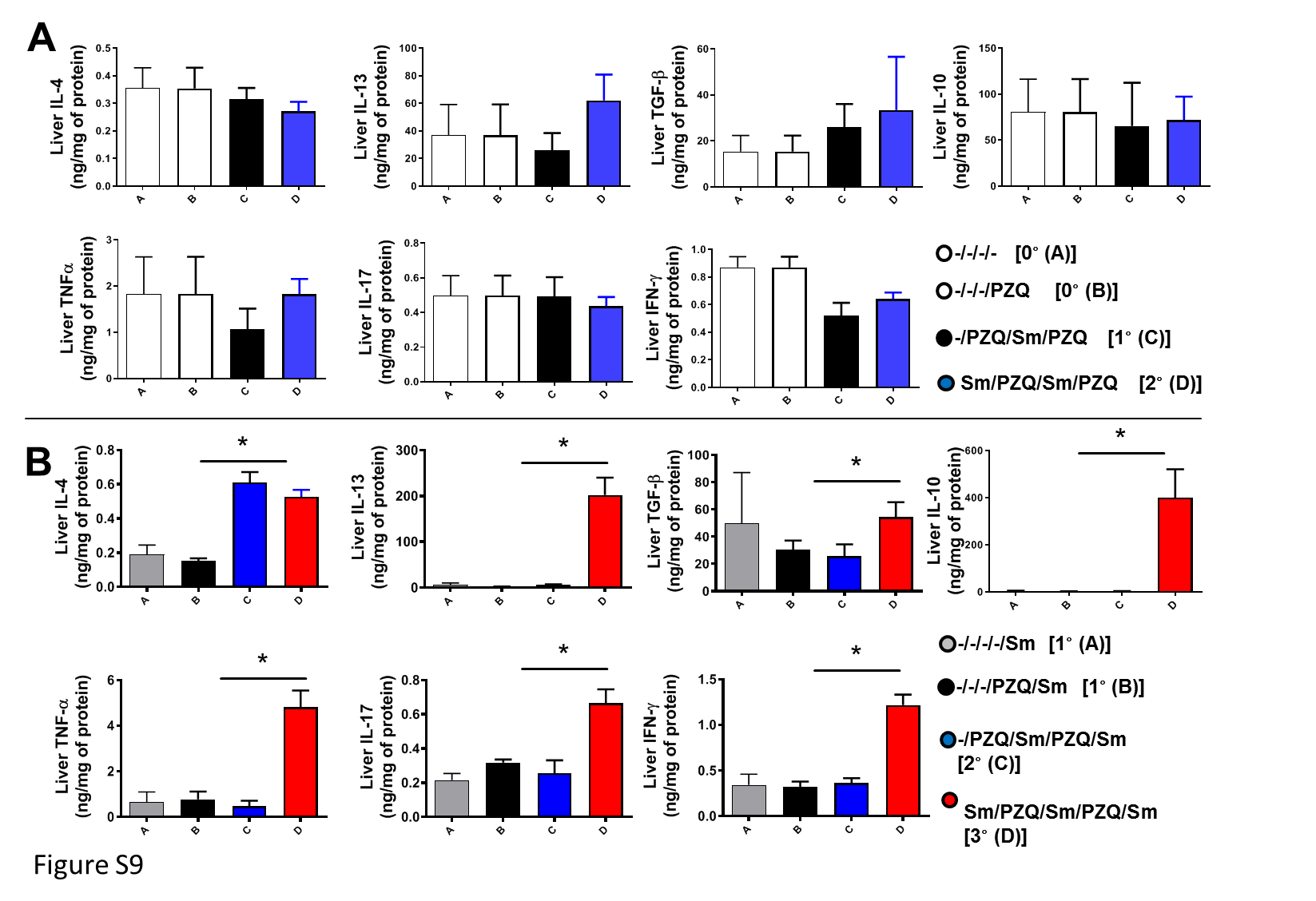
Figure S9: Liver cytokine production after repeated cycles of *S. mansoni* infection and PZQ treatments. Outcome measure = Change in immune markers in animals.** Liver lobes collected from animals treated according to the differential scheme A, B, C, D and sacrificed either at week 15 (A, i.e. P1) or week 25 (B, i.e. P2) were homogenized and the production of cytokines (IL-4, IL13, TGF-b, IL-10, TNF-a, IL-17, IFN-g) was determined as a function of the protein amount of liver tissue probed. Data shown are means from one of 2 experiments, with 4-7 mice per group in each experiment. NS = p > 0.05; * = p < 0.05; ** = p < 0.01; *** =, p < 0.001; **** = p < 0.0001 as determined by Kruskal-Wallis test with correction for multiple comparisons by Dunn’s test. Significant salient comparisons between groups are displayed.
